# Determination of the phloem transport pathways and destination of photosynthates in soybean using autoradiography and fluorescent tracer imaging

**DOI:** 10.1080/15592324.2025.2552897

**Published:** 2025-08-29

**Authors:** Ai Kaiho-Soma, Yuko Kurita, Natsuko I. Kobayashi, Tomoko M. Nakanishi, Keitaro Tanoi

**Affiliations:** a Graduate School of Agricultural and Life Science, The University of Tokyo, Tokyo, Japan; b Fukushima Institute for Research, Education and Innovation (F-REI), Fukushima, Japan

**Keywords:** Autoradiography, soybean, photosynthate, phloem, translocation

## Abstract

Vascular tissues transport water and nutrients in plants, with the phloem distributing photosynthates from source to sink. The direction of phloem transport is determined by the positional relationship between sources and sinks and by vascular connections. Although aspects of phloem transport have been studied, a comprehensive understanding remains lacking. Here, we used soybean as a model system to investigate the translocation pathways and destinations of photosynthates using autoradiography with ¹⁴C-labeled sucrose and fluorescent imaging with carboxyfluorescein (CF), a known phloem tracer. Soybean exhibits simple phyllotaxy, with alternate trifoliate leaves arranged oppositely along the stem. Applying ¹⁴C-sucrose to mature leaves revealed that young developing leaves received photosynthates from source leaves on both sides of the stem. To visualize pathways, ¹⁴C-sucrose and carboxyfluorescein diacetate (CFDA) were applied to sequential source leaves. Signals from ¹⁴C and CF in the stem's vascular bundles showed no overlap, indicating distinct transport pathways. Additionally, when ¹⁴C-sucrose was applied separately to the left and right halves of a single mature leaf, it was followed corresponding sides to the sink leaves. These findings demonstrate that photosynthates are delivered to sink tissues via multiple, well-compartmentalized phloem pathways, providing new insight into the spatial organization of phloem transport.

## Introduction

Vascular tissues are essential for the transport of water and nutrients in plants. The primary components of vascular tissues are xylem and phloem. Xylem is responsible for the unidirectional transport of water and dissolved minerals from roots to leaves. In contrast, phloem transports metabolic products, amino acids, hormones, ions, mRNAs, and various other substances. Among these, the most studied are the photosynthates, which are translocated from mature source leaves to developing sink organs. Mature leaves, which produce more photosynthates than their require, act as sources, exporting the surplus to nutrient-demanding sinks for growth and maturation. Unlike xylem transport, phloem transport is bidirectional and depends on the relative positions of source and sink organs. Downward transport occurs from upper sources to lower sinks, and upward transport occurs from lower sources to upper sinks. In addition, the distance between source and sink influences allocation patterns: lower sources preferentially supply nearby lower sinks, and upper sources preferentially supply nearby upper sinks.^1^

The arrangement of leaves on the stem, known as phyllotaxis, is closely related to vascular connections and determines the destinations of photosynthates.^2^ The relationship between phyllotaxis and source-sink translocation patterns has been reported in model plants such as Arabidopsis^3^ and tobacco,^4^ as well as in non-model plants such as sour cherry.^5^ In tomato, detailed studies have been reported on phyllotaxis, vascular connections, and their relationship to photosynthate translocation to fruits.^6^^,^^7^ These studies have shown that photosynthates translocated from a given source leaf are preferentially delivered to sink organs located on the same side of the stem. However, these analyses have mostly focused on unidirectional, upward translocation from a single lower source leaf to upper sinks. The mechanism underlying bidirectional translocation, and how multiple source leaves contribute to or coordinate this process, remains largely unresolved. In addition, although translocation pathways have been inferred from phyllotaxis, the actual pathways have not been clearly identified in most species.

The aim of the present study was to improve our understanding of comprehensive phloem transport by investigating the translocation pathways, directions and destinations of photosynthates using soybean plants. Soybean (*Glycine max*, Fabaceae family), an annual dicotyledonous plant, exhibits simple phyllotaxy. In the initial stages of development, the soybean plant forms a pair of cotyledons, followed by a pair of primary leaves (PL) arranged at right angles to the cotyledons. Subsequently, trifoliate leaves, each consisting of three leaflets, develop in sequence, alternating and positioned perpendicularly to the PL, each opening at an angle of 180°.^8^ These trifoliate leaves are numbered sequentially in the order in which they emerge, and are referred to as L1, L2, L3, and so on. Leaves at positions *n*, n + 2, and n + 4 are arranged on the opposite side of the stem relative to those at positions n + 1, n + 3, and n + 5. To examine phloem translocation, ^14^C-sucrose and 5′(6)-carboxyfluorescein diacetate (CFDA) were applied to source leaves at different positions in the same plant, and their distribution in the stem was visually analyzed. CFDA is widely used as a phloem-mobile tracer, as its fluorescent product, carboxyfluorescein (CF), moves symplastically and remains confined to the phloem region.^9^ Notably, we uncovered distinct and compartmentalized phloem transport pathways depending on whether the source leaves were positioned on opposite sides of the stem or whether the left or right half of a single leaf was used. Based on these findings, we propose a more refined model of phloem transport and unloading than those inferred from previous observations.

## Materials and methods

### Plant material and growth conditions

Seeds of soybean (Glycine max. cv. Kosuzu) (Sato Masayuki Seed Company, Iwate, Japan) were sown on moist vermiculite and germinated in the dark at 27°C. The germinated seedlings were then transferred to 1/2 Hoagland hydroponic solution. The plants were grown under a 16 h light/8 h dark cycle (280 μmol m^−2^ s ^−1^) at 27°C. The hydroponic solution was refreshed every week, and the plants were hydroponically cultivated for 8 to 14 d. To maintain experimental consistency, plant samples were selected based on their growth stage rather than the number of days of culture. To investigate the destination of photosynthate, we focused on sink leaves. In cases where L2 was expanding, the developmental state of L3 was used as an indicator. We distinguished between the folded and unfolding states of L3, and referred to the conditions as “L2-expanding” and “L3-unfolding”. To investigate the translocation pathways, more than 80% of the expanded leaves were selected as the source leaves.

### Application of ^14^C-sucrose and 5’ (6)-carboxyfluorescein diacetate (CFDA)

The selected leaves were gently abraded with sandpaper, followed by the application of ^14^C-sucrose (7.4 MBq/ml, 5 μl) (MC266, Moravek) and/or CFDA (1 mM, 5 μl) (19582, CAY). The leaf was then covered with polyethylene film to prevent desiccation. The plants were cultivated under growth conditions for 4 to 6 h for the translocation pathway analysis, and 22 to 24 h for identification of the destination.

### Imaging radioisotopes and fluorescent chemicals

^14^C imaging: The distribution of the ^14^C signal in soybean plants was visualized by autoradiography. For detecting the ^14^C signal in the plants, samples were covered with polyethylene film and exposed to an imaging plate (BAS IP MS 2040, FUJIFILM) within a cassette at 4°C for 1 to 3 d, depending on the ^14^C activity. For in the stem analysis, freehand cross-sections were prepared using a razor blade. The sections were cleaned of exudates and wrapped between a 1.2 μm thick polyphenylene sulphide film and cryofilm (Cryofilm Type 2C, Leica) to prevent drying and radioisotope contamination. These sections were then exposed to a high-sensitive imaging plate (BAS-TR2040, FUJIFILLM) within a cassette at −80°C for 9 to 14 d, depending on the ^14^C activity. The resulting ^14^C activity images were acquired using fluorescent image analyzer (Amersham Typhoon, Cytiva).

CF imaging and bright-field (BF) imaging: Before exposure to the imaging plate for ^14^C imaging, CF and BF images of wrapped stem cross-sections were captured using a fluorescence microscope (BX60, Olympus). The entire process, from section preparation to storage at −80°C took less than an hour. To identify the structure, cross-sections that did not contain ^14^C or CF were stained with **0.01%** (w/w) toluidine blue (TB) and observed under a microscope.

Imaging analysis: These images were analyzed using ImageJ (version 1.53t). To support the understanding of signal distributions, each image in the main stem section was set with the L1 petiole connecting to the left, and the signal around the cambium was captured in a 360° clockwise direction starting from the 12:00 position. The signal intensities of ^14^C and CF are expressed as relative values ranging from 0 to 1. Signal differences represent the CF intensity minus the ^14^C intensity, ranging from −1 to 1. The signal differences will be close to 0 throughout if the distributions of the ^14^C and CF intensities match. Imaging analyses were performed on more than three individual soybeans, and comparable results were obtained. Representative results are shown in each figure.

## Results

### Translocation of photosynthates from mature leaves at different leaf positions

**Figure 1.**
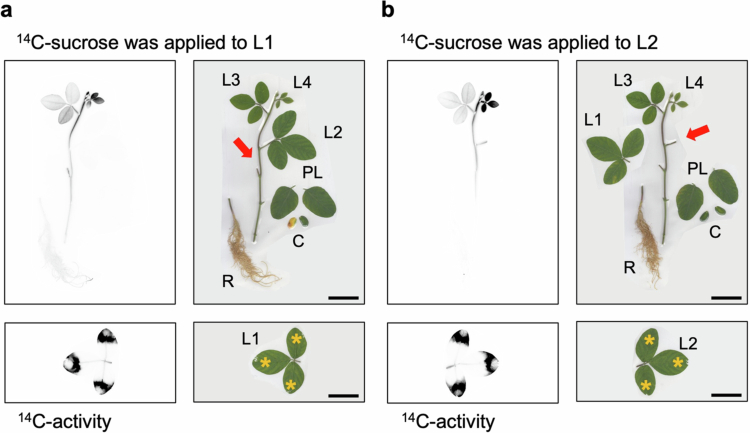
Destination of photosynthates from mature leaves at different leaf positions. The figures present autoradiography images and corresponding photographs. ^14^C-Sucrose was applied to L1 (a) or L2 (b) of the soybeans at L4-unfolding stage (11 d after emergence (DAE)). The red arrows indicate where the ^14^C-treated leaf was connected. Asterisks indicate where ^14^C-sucrose was applied. Both results were similar for each of the six individuals. Scale bar: 5 cm. L1: first trifoliate; L2: second trifoliate; L3: third trifoliate; L4: fourth trifoliate; PL: primary leaf; C: cotyledon; R: root.

To identify the destination of photosynthates derived from individual source leaves, ^14^C-sucrose was applied to either the L1 or L2 of soybean plants at the L4-unfolding stage (Figure 1). In the leaves where ^14^C-sucrose was applied, extremely high ^14^C signal was found in the area where the ^14^C-sucrose solution was added, while less ^14^C signal was detected at the midvein and no detectable activity elsewhere on the leaf. Other than the treated leaf, high ^14^C activity at L4, moderate activity at L3, and little ^14^C activity in the PL and cotyledons was detected regardless of whether L1 or L2 was treated with ^14^C. When ^14^C-sucrose was added to L1, L2 exhibited negligible ^14^C activity. When ^14^C-sucrose was added to L2, L1 showed minimal ^14^C activity. In the stem, higher ^14^C activity was observed in the upper part, including the shoot apex, in both plants with ^14^C-sucrose applied to L1 and L2. Although ^14^C activity was detected below the node of ^14^C-sucrose-added leaf, the activity levels were relatively low. The signal of ^14^C in the roots was detected only when ^14^C-sucrose was applied to L1.

### Behavior of ^14^C-sucrose and CF in the stem

**Figure 2.**
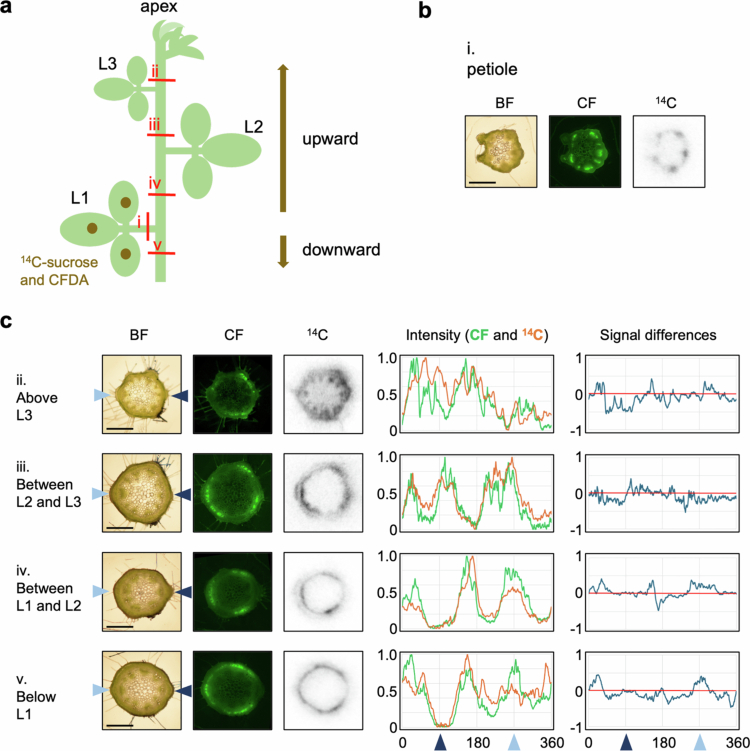
Distribution of ^14^C-sucrose and CF in the stem and petiole. (a) Schematic representation of the simultaneous application of ^14^C-sucrose and CFDA to L1 of soybean at stage when L2 was more than 80% expanded (12 DAE). The red line indicates the sectioned area subjected to imaging analysis. Consistent results were obtained across more than four samples. (b) Image data of petiole sections as shown in (a). (c) Images and corresponding analytical data of internode sections from (a). Image data were arranged from left to right: BF, CF fluorescence, and ^14^C autoradiograph. Arrowheads on either side of the BF denote the direction of the petiole connection, with the left aqua color indicating the L1 connection direction and the right navy color indicating the L2 connection direction. The graphs present the analytical data. The intensity represents a graph of the relative CF fluorescence intensity (green line) and ^14^C activity (orange line) around the cambium. Signal differences refer to the CF intensity subtracted from the ^14^C intensity (red line: 0 value). The arrowheads on the horizontal axis of the graph correspond to the position of the arrowhead on the BF image. Scale bar: 1 mm. L1: first trifoliate; L2: second trifoliate; L3: third trifoliate; BF: bright-field; CFDA: carboxyfluorescein diacetate; CF: carboxyfluorescein.

To confirm that ^14^C-sucrose and CF exhibit similar behavior in the phloem, a mixture of ^14^C-sucrose and CFDA was applied to L1 of the soybean plants at the stage when L2 was more than 80% expanded. Cross-sections of the petiole and main stem were prepared to observe the CF and ^14^C distributions (Figure 2). In the petiole of the leaves to which tracers were applied, both ^14^C and CF signals were detected throughout the vascular bundles. In the stem, CF intensities patterns were as follows: Three large peaks were displayed at internode between L2 and L3, between L1 and L2, and below L1. Notably, the signals observed between L1 and L2, and below L1 were located at the same regions on the stem (around 30°, 150° and 270°), even though the flow directions from L1 were opposite. Above L3, two peaks were observed, which seemed to result from the merging of the three peaks.

**Figure 3.**
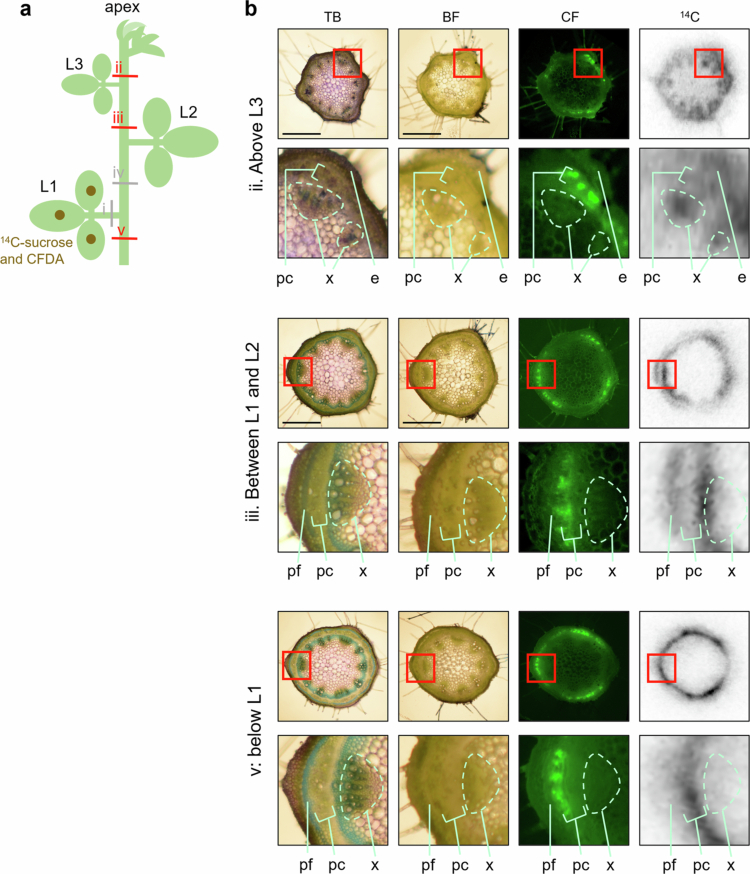
TB-stained sections of the stem. Sections corresponding to those in Figure 2c-ii, iii and v were stained with TB. Consistent results were obtained across more than six samples. (a) Schematic representation of the experiment shown in Figure 2. (b) Image data of internode sections shown in (a), arranged from left to right: TB, BF, CF fluorescence and ^14^C autoradiograph. The lower panels show enlarged views of upper panels with the area enclosed in red magnified fourfold. CF and ^14^C images are the same as in Figure 2. Scale bar: 1 mm. e: epidermis; pc: phloem and cambium; pf: phloem fiber; x: xylem; L1: first trifoliate; L2: second trifoliate; L3: third trifoliate; BF: bright-field; CFDA: carboxyfluorescein diacetate; CF: carboxyfluorescein; TB: toluidine blue.

To clarify the exact location of ^14^C detection, ^14^C autoradiography and TB-stained images were compared (Figure 3). Although perfect identification was not possible because the images were not derived from the same sample, CF signals were only seen in the phloem in all cases. Above L3, ^14^C signals were observed not only in the phloem but also in the xylem and epidermis. At other parts, it is difficult to determine whether ^14^C signals were in the cambium and/or phloem. Occasionally, ^14^C signals were also detected in the phloem fibers. In any case, the signals of ^14^C and CF intensities showed similar patterns (Figure 2c). These data indicate that ^14^C and CF passed through the same regions of the vascular bundles.

### Translocation pathways of photosynthates from mature leaves at different leaf positions

**Figure 4.**
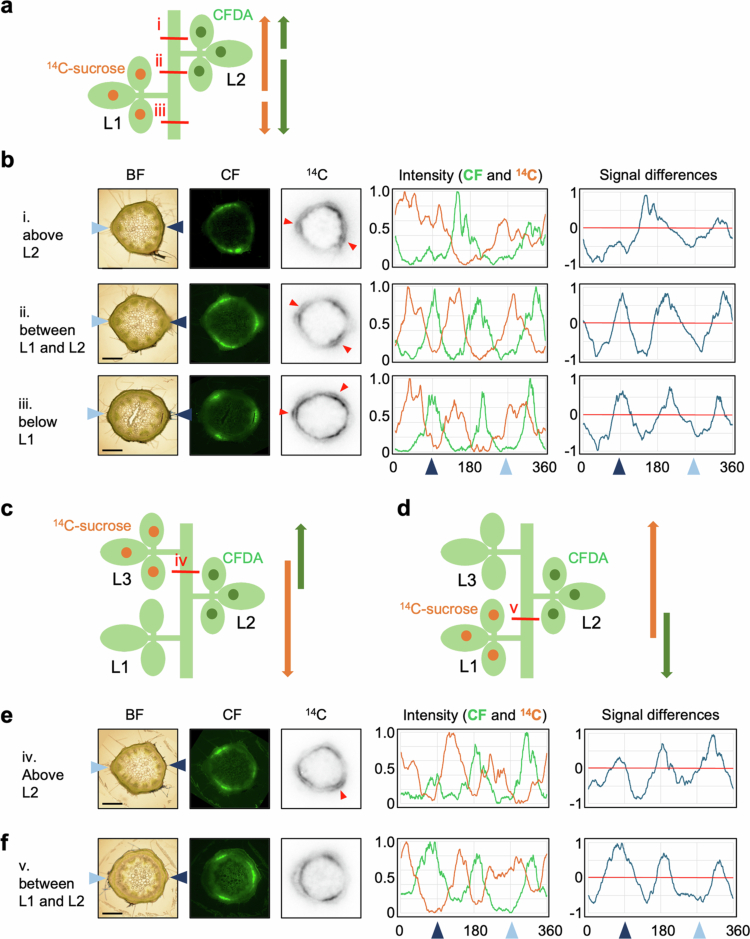
Translocation pathways of photosynthates from mature leaves at different leaf positions. (a, c, d) Schematic representation of the simultaneous application of ^14^C-sucrose and CFDA. (a) ^14^C-sucrose and CFDA were applied to L1 and L2 of soybeans at the stage when L2 was more than 80% expanded (12 DAE), respectively. CFDA was applied to L2, and ^14^C-sucrose was applied to L3 (c) or L1 (d) of soybeans at the stage when L3 was more than 80% expanded (14 DAE). The red line indicates the sectioned area subjected to imaging analysis. The arrows indicate the direction of signal translocation, which orange indicating ^14^C and green indicating CF. (b, e, f) The figure presents images and corresponding analytical data of stem sections, as shown in (a, c, d), respectively. Consistent results were obtained for seven (b) and three (e, f) individuals, respectively. Image data were arranged from left to right: BF, CF fluorescence, and ^14^C autoradiograph. Arrowheads on either side of the BF denote the direction of the petiole connection, with the left aqua color indicating the L1 connection direction and the right navy color indicating the L2 connection direction. Red arrowheads in the ^14^C image highlight regions where signals were detected in the phloem fibers. The graphs present the analytical data. The intensity represents a graph of the relative CF intensity (green line) and ^14^C activity (orange line) around the cambium. Signal differences refer to the CF intensity subtracted from the ^14^C intensity (red line: 0 value). The arrowheads on the horizontal axis of the graph correspond to the position of the arrowhead on the BF image. Scale bar: 1 mm. L1: first trifoliate; L2: second trifoliate; L3: third trifoliate; BF: bright-field; CFDA: carboxyfluorescein diacetate; CF: carboxyfluorescein.

**Figure 5.**
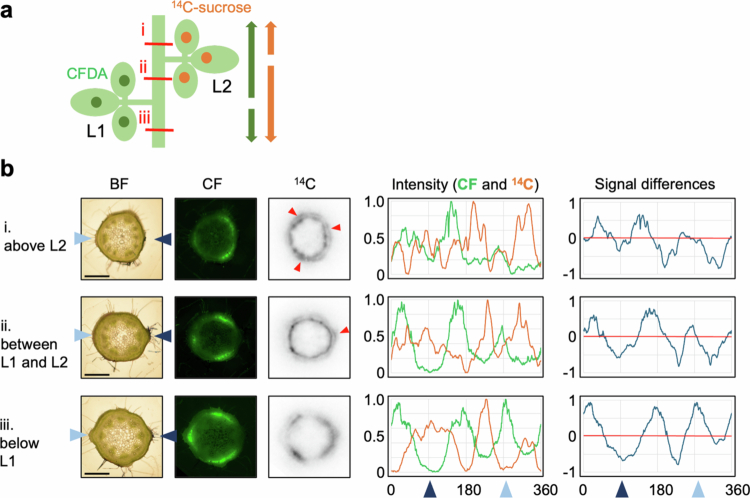
Confirmation experiment with marker switching. (a) Schematic representation of swapped applications of CFDA and ^14^C-sucrose in Figure 4a and b. CFDA applied to L1 and ^14^C-sucrose applied to L2 of soybean at stage when L2 was more than 80% expanded (12 DAE). Consistent results were obtained for four individuals. The red line indicates the sectioned area subjected to imaging analysis. The arrows indicate the direction of signal translocation, which orange indicating ^14^C and green indicating CF. (b) The figure presents images and corresponding analytical data of stem sections, as shown in (a). Image data were arranged from left to right: BF, CF fluorescence, and ^14^C autoradiograph. Arrowheads on either side of the BF denote the direction of the petiole connection, with the left aqua color indicating the L1 connection direction and the right navy color indicating the L2 connection direction. Red arrowheads in the ^14^C image highlight regions where signals were detected in the phloem fibers. The graphs present the analytical data. The intensity represents a graph of the relative CF intensity (green line) and ^14^C activity (orange line) around the cambium. Signal differences refer to the CF intensity subtracted from the ^14^C intensity (red line: 0 value). The arrowheads on the horizontal axis of the graph correspond to the position of the arrowhead on the BF image. Scale bar: 1 mm. L1: first trifoliate; L2: second trifoliate; BF: bright-field; CFDA: carboxyfluorescein diacetate; CF: carboxyfluorescein.

Photosynthates were translocated from different sources to the same sink leaves (Figure 1). To confirm the translocation pathways, ^14^C-sucrose was applied to L1, and CFDA was applied to L2 in soybeans at the stage when L2 was more than 80% expanded (Figure 4a and b). Imaging analysis was subsequently conducted at each position. At the internodes between PL and L1, and between L1 and L2, both ^14^C and CF intensities exhibited three peaks in the same regions, respectively. In these two positions, CF should be transported downward. In contrast, the direction of ^14^C translocation was upward between L1 to L2, and downward between PL and L1. At the internode between L2 and L3, the ^14^C and CF intensities exhibited two peaks similar to those shown in Figure 2c-ii. At all internodes, the peaks of ^14^C and CF did not overlap. These findings were confirmed by similar experiments in which the positions of ^14^C-sucrose and CFDA leaf applications were swapped (Figure 5). These results demonstrate that the pathways that the photosynthates coming from L1 and that from L2 take may not be the same.

L1 and L2 are located on opposite sides of the stem, which may account for the distinct vascular regions utilized within the stem. To verify the behavior of translocation from leaves located on same side to the stem, we compared the translocation pathways from L1 and L3. Imaging of stem sections was performed by foliar application of ^14^C-sucrose and CFDA to L1 and L3, respectively, at the stage when L3 was more than 80% expanded. However, ^14^C and CF signals could not be clearly detected in the same sections probably because the distance between L1 and L3 are too long (data not shown). Therefore, we divided the experiment into two parts, one comparing the translocation pathways from L2 and L3 and the other from L1 and L2 (Figure 4c–f). Different plants at the same growth stage were used. For both plants, CFDA was added to L2, while ^14^C-sucrose was added to L3 or L1. In each experiment, clear signals of both ^14^C and CF were detected only in the internode between the two leaves with applied CFDA and ^14^C-sucrose, respectively. In both images, the signals of ^14^C and CF intensities showed three peaks, and peaks were clearly separated.

### Translocation pathways of photosynthates from different areas within a single source leaf

**Figure 6.**
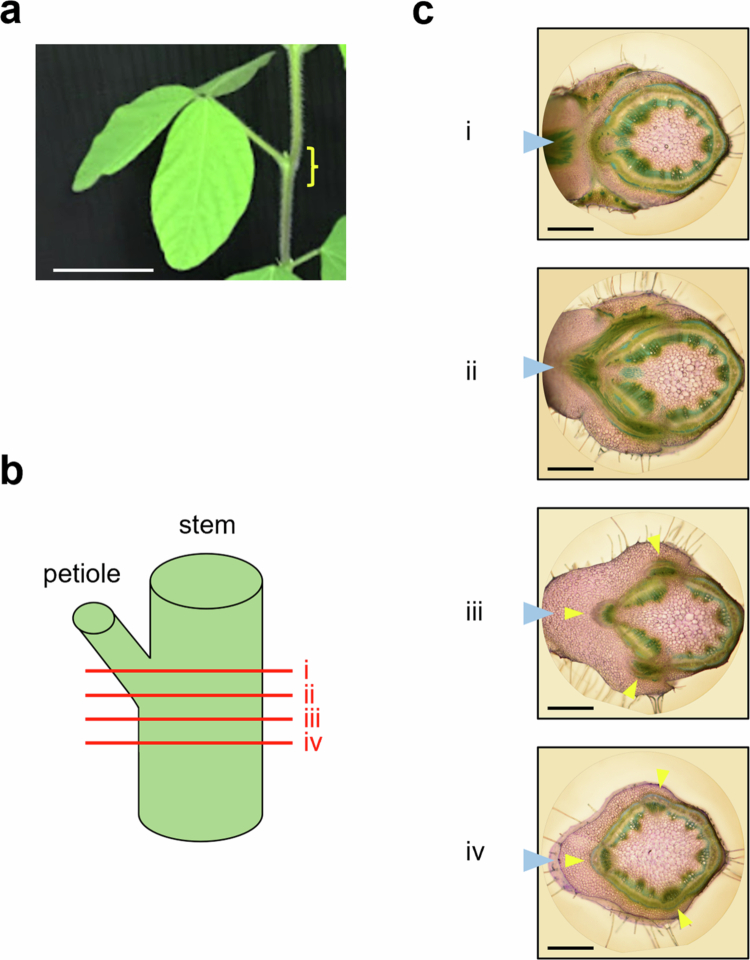
TB-stained sections of the node. (a) Photograph of a soybean focusing on L1. Cross sections of soybeans at stage when L1 was more than 80% expanded (10 to 12 DAE) were stained with TB. (b) Schematic representation of the cross sections location. The red line indicates the sectioned area subjected to imaging analysis. Consistent results were obtained from more than five samples. (c) TB-stained image data shown in (b). Aqua color arrowheads on the left side of the image denote the direction of the L1 petiole connection. Yellow arrowheads in images iii and iv highlight regions where vascular bundles from the petiole enter the stem. Scale bars: 5 cm in (a); 1 mm in (c).

**Figure 7.**
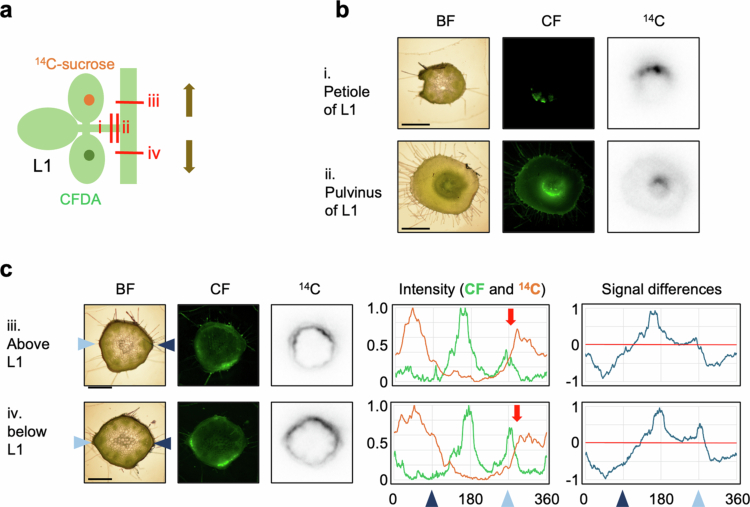
Translocation pathways of photosynthates from different areas within a single source leaf. (a) Schematic representation of the experiment conducted. ^14^C-Sucrose was applied to the right lateral leaflet of L1, while CFDA was applied to the left lateral leaflet of L1 of the soybean at the stage when L1 was more than 80% expanded (9 to 12 DAE). The red line indicates the sectioned area subjected to imaging analysis. Arrows denote the direction of signal translocation. Consistent results were obtained for five samples. (b) Images of petiole and pulvinus sections as shown in (a). (c) Images and corresponding analytical data of the stem sections as shown in (a). Image data were arranged from left to right: BF, CF fluorescence, and ^14^C autoradiograph. Arrowheads on either side of the BF denote the direction of the petiole connection, with the left aqua color indicating the L1 connection direction and the right navy color indicating the L2 connection direction. The graphs present the analytical data. The intensity represents a graph of the relative CF fluorescence intensity (green line) and ^14^C activity (orange line) around the cambium. Signal differences refer to the CF intensity subtracted from the ^14^C intensity (red line: 0 value). The arrowheads on the horizontal axis of the graph correspond to the position of the arrowhead on the BF image. The red arrows in the intensity graph denote around 270° region. Scale bar: 1 mm. L1: first trifoliate; BF: bright-field; CFDA: carboxyfluorescein diacetate; CF: carboxyfluorescein.

In these tracer experiments, both ^14^C and CF intensities showed three peaks in stem cross-sections near the node of the tracer-applied leaf (Figures 2c–iii, iv, v, 4b-ii, iii, e, f, 5b–ii and iii). TB-stained images showed that the vascular bundles entering the stem from the petiole divided into three branches before merging (Figure 6). The trifoliate of soybeans consists of three leaflets, one terminal leaflet and two lateral leaflets. To determine whether these three vascular pathways correspond to the individual leaflets, ^14^C-sucrose and CFDA were applied to the right and left lateral leaflets of L1 at the stage when L1 was more than 80% expanded, and imaging analysis was subsequently conducted. As a result, the distributions of ^14^C and CF in the petioles and pulvinus were clearly distinct (Figure 7). In the stem, both ^14^C and CF signals showed two peaks in the same regions of the internode between L1 and L2, and between PL and L1, even when the translocation directions were opposite (upward vs. downward). Both ^14^C and CF signals showed high intensities around 270°, but the peak did not overlap. These results suggest that photosynthates loaded from different areas within the same source leaf generally follow distinct translocation pathways, though partial overlap may occur.

### Destination of photosynthates from different areas within a single leaf

**Figure 8.**
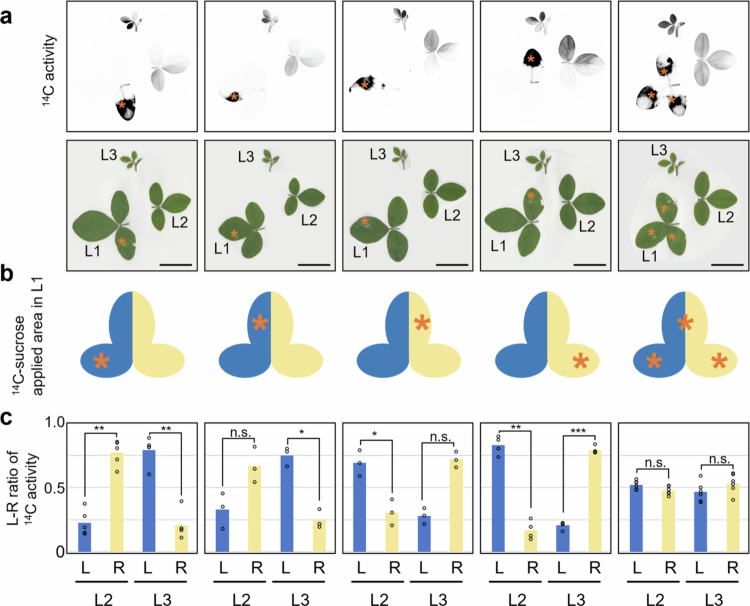
Destination of photosynthates from different areas within a single leaf. ^14^C-Sucrose was added to a specific area of L1 of the soybeans at L3-unfolding stage (9−10 DAE). Consistent results were obtained for three to five individuals in each condition. (a) The figures present autoradiography images and corresponding photographs. (b) Schematic representation of L1. The left side is colored blue, and the right side is colored yellow. The orange asterisks indicate where ^14^C-sucrose was applied. (c) The left-right ratio of ^14^C activity in one trifoliate of L2 and L3. Statistical significance was assessed by Paired *t*-test. *P < 0.05; **P < 0.01; ***P < 0.001. *n*.s.: not significant. Scale bar: 5 cm. L; light; R: right; L1: first trifoliate; L2: second trifoliate; L3: third trifoliate.

Within a single source leaf, different areas utilized different translocation pathways (Figure 7). To investigate the destinations from each area, ^14^C-sucrose was applied either to one lateral leaflet or to either side of the terminal leaflet of L1 at the L3-unfolding stage (Figure 8). When ^14^C-sucrose was applied to the left lateral leaflet, high ^14^C signal was observed on the right side of L2 and the left side of L3. Conversely, when ^14^C-sucrose was applied to the right lateral leaflet, high ^14^C signals was observed on the left side of L2 and the right side of L3. When ^14^C-sucrose was applied separately to each side of the terminal leaflet, L2 and L3 showed an uneven distribution of ^14^C activity, similar to the pattern observed when ^14^C-sucrose was applied to either lateral leaflet. These results show that the destination of ^14^C depends on the ^14^C-sucrose application area within the source leaf, specifically whether ^14^C-sucrose was applied to the left or right side.

## Discussion

**Figure 9.**
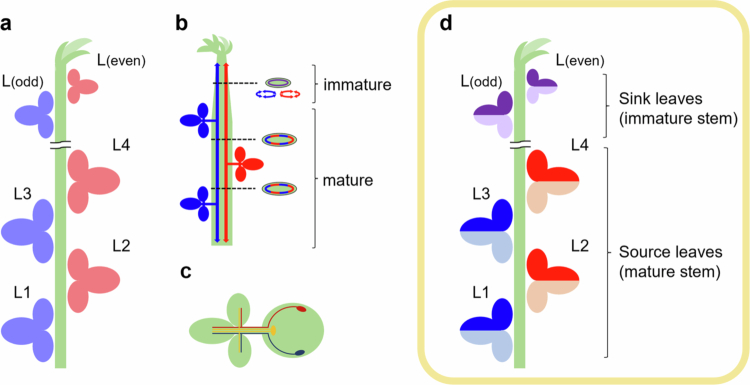
Models for the translocation pathways and destination of photosynthates in a soybean plant. (a) Speculative model of phloem loading and unloading (commonly accepted). Leaves located on the same orthostichy share a common vascular bundle, and photosynthates are thought to be translocated from source leaves to sink leaves located on the same side. (b) Translocation pathways in the stem summarized from Figures 2, 4 and 5. Source leaves located on the same orthostichy share a common vascular bundle regardless of direction. Source leaves on the opposite side use different translocation pathways, but it is thought that in the upper stem, these regions spread and share with each other. (c) Translocation pathways from different regions of one leave predicted from Figures 6, 7 and 8. Each leaf has three major vascular bundles that connect to the stem. These bundles are functionally divided between the left and right halves of the leaf. (d) Updated model of phloem loading and unloading based on our results. Source leaves located on the same orthostichy share the same vascular bundle, but photosynthates are now understood to be translocated from source leaves to sink leaves located on both sides. The loading and unloading of photosynthates occur on the same lateral side in leaves, indicating a division of the leaf into left and right segments. L1: first trifoliate; L2: second trifoliate; L3: third trifoliate; L4: fourth trifoliate.

Photosynthates translocation in plants follows specific vascular pathways, which are associated with phyllotaxy – the arrangement of leaves on a stem. In soybean, which exhibits a simple two-ranked phyllotaxy, it has been inferred that photosynthates are translocated along the same side of the source leaves^10–13^ (see Figure 9a). For example, Biddulph et al. reported that ^32^*P* introduced into the L1 of soybean was translocated within the phloem of vascular bundles associated with the L3.^11^ In reproductive-phase soybean, photosynthates from specific leaves are distributed to pods on the same node and on alternate nodes.^10^^,^^13^ However, the detailed translocation pathway has not yet been fully elucidated.

To address this knowledge gap, we employed dual labeling with a radioisotope (^14^C) and CF, enabling visualization of translocation pathways in the same plant tissue. Radioisotope tracing has a long history in the analysis of phloem translocation. Biddulph and Cory investigated the translocation pathways from different leaf positions in red kidney bean seedlings by supplying ^14^CO_2_ to L1 and NaH_2_^32^PO_4_ to L2, detecting radioisotope activity at the internode between L1 and L2.^14^ However, in their experiments, the signals from ^14^C and ^32^*P* could not be clearly distinguished. Later, using separate applications of ^14^CO_2_ to either L1 or L2, they found that different sets of vascular bundles were involved in translocation from each leaf.^15^ In the present study, we successfully extended these findings by directly visualizing the dual-labeled pathways in cross-sections of the same sample (Figures 4 and 5).

It is important to note, however, that CF was found to be unsuitable for analyzing long-distance translocation in well-developed plants. When soybean had reached a stage where L3 was more than 80% expanded, CF signals from L3 and ^14^C signals from L1 could not be clearly observed in the same section. Because CF is mainly transported via simple diffusion, its movement is limited to nearby tissues and becomes significantly diluted as the plant grows and internodes elongate, making it increasingly difficult to detect in distal tissues. On the other hand, at this developmental stage, ^14^C-sucrose applied to L1 was predominantly translocated downwards rather than upward. This is because the lower sink organs, such as the roots, are spatially closer to L1 than the upper sinks like the shoot apex or young leaves. As a result of both the limited transport range of CF and the downward bias in ^14^C translocation, it was not possible to obtain a section in which both CF and ^14^C signals were clearly detected. This detailed comparing analysis was possible because the experiments were conducted during the early stage of vegetative growth.

Near the shoot apex, CF signals were restricted to the phloem, whereas ^14^C signals were also detected in the xylem, and epidermis in addition to phloem. Since CF moves symplastically through plasmodesmata,^16^ whereas ^14^C can be unloaded through additional pathways beyond plasmodesmata. Similar ^14^C distribution in the xylem of young stems has been reported following ^14^CO_2_ application to source leaves.^4^^,^^11^​​​​​ In contrast, in the internodes between mature leaves, ^14^C was primarily observed in the regions corresponding to phloem and/or cambium (Figures 2–5). Occasionally, ^14^C signals were also detected in the phloem fibers (Figures 2–5), which are structural support tissues. These results suggest that, while the phloem serves as the main conduit for long-distance translocation, the stem may also unload photosynthates for its own growth and structural maintenance. Notably, the ^14^C signal peaks were slightly broader than those of CF (Figure 2c-ii and v), indicating lateral movement of photosynthates after unloading.

To understand where photosynthates are distributed throughout the plant, the destination of photosynthates from source leaves located on opposite sides of the stem was investigated (Figure 1). We found that both L1 and L2 applied photosynthates to the same sink leaves, L3 and L4. Interestingly, weak ^14^C activity was detected in the roots only when ^14^C-sucrose was added to L1. This is likely because the roots acts as weak sinks compared to young immature leaves, and L1 is anatomically closer to the roots than L2. Among the sinks, L4 exhibited higher ^14^C activity than L3, indicating greater sink strength.

Cross-section imaging revealed that photosynthates from source leaves on opposite sides of the stem were translocated through distinct phloem pathways, utilizing alternating sectors among the six divided vascular regions in the stem (Figures 2, 4 and 5). This is likely due to the anatomical structure in which each vascular bundle from the petiole splits into three, which then connect to vascular bundle in the stem (Figure 6). Thaine et al. also reported that the leaf vasculature in soybean connect to three wide bundles that enter the stem.^1^ Thus, leaves positioned along the same orthostichy share the same vascular bundle regions, whereas the leaves on opposite side utilize different phloem pathways (Figure 4). These results are consistent with commonly accepted model (Figure 9a). However, in our experimental results, L3 and L4 received photosynthates from both L1 and L2. This may be due to immaturity of vascular tissues in the upper stem where these sink leaves are located, as evidenced by broad and overlapping tracers' peaks in these regions (Figures 2 c-ii, 4b-i and 5b-i). In young growing leaves, plasmodesmata in the major veins are gradually lost or narrowed, which hinders unloading.^17^ A similar process may occur in the stem, where plasmodesmata-mediated transport prominates in the younger stem. Furthermore, by organizing the vascular bundles into three distinct sectors that alternate around the stem, rather than a simple splitting them into two halves, it may be possible to share translocation pathways partially between leaves on opposite sides. Such an arrangement could enable sink leaves to receive phloem-transported materials from multiple source leaves, as indicated by the observed partial overlap in transport pathways.

Imaging analysis of stem cross sections was conducted both above and below the tracer-applied leaves. The sections above the application site revealed upward translocation toward apical sinks, while the sections below showed downward translocation toward basal sinks (e.g., roots). In both directions, the tracer signals were distributed similarly, showing three distinct intensity peaks, regardless of the translocation direction (Figures 2c-iv and v, 4b-ii and iii of ^14^C, and 5b-ii and iii of CF). These results suggest that the same vascular bundle regions were utilized for translocation in both directions. A summary of the translocation pathways is shown in Figure 9b. So far, it remains unclear whether bidirectional movement occurs within the same vascular bundle via separate sieve tube or temporally alternating use of the same sieve tubes. To resolve the mechanisms, further high-resolution analyses capable of distinguishing individual sieve tubes are needed. The mechanism underlying long-distance translocation in phloem is explained by the pressure flow hypothesis, proposed by Münch,^18^ where the translocation of substances is driven by osmotic pressure gradients between sources and sinks. Knoblauch et al. provide strong support for this hypothesis by measuring several parameters of sieve tube transport in morning glory, but their study focused on unidirectional flow after pruning sink tissues.^19^ Further investigation is needed to determine how bidirectional translocation is regulated within the same vascular region, and whether distinct sieve tubes or temporal alternation are involved in accommodating flow in both directions.

A unique finding of our study is the consistent detection of left–right asymmetry in ^14^C activity within individual leaves. Although uneven ^14^C distribution has been reported in some species (e.g., Cucurbita pepo, sugar beet and tobacco), these studies generally described base-tip differences that were attributed to developmental gradients.^20^^,^^21^ To our knowledge, this is the first report to demonstrate a consistent pattern of left-right asymmetric translocation from leaves and to trace the corresponding translocation pathways. Our data so that each lateral leaflet utilizes two of the three distinct vascular regions in the stem with partial sharing between regions (Figure 7). Even in the terminal leaflet, translocation from the left and right sides results in different distribution patterns within the sink leaves (Figure 8). Based on these findings, we propose a model in which each leaf is functionally divided into left and right halves, each utilizing a separate vascular pathway (Figure 9c). This functional compartmentalization of phloem loading sites may help limit the spread of local damage or infection, thereby providing physiological robustness.

The destination of photosynthates, revealed by our experiments, is summarized in Figure 9d. This model is consistent with previous reports showing that specific leaves supply pods at the same and alternate nodes,^10^^,^^13^ as the stems connected to pods are already mature. However, further studies are needed to experimentally verify this pattern under the present conditions.

In Summary, our study provides deeper insights into the destinations and translocation pathways of photosynthates in soybean. Despite its simple phyllotaxy, soybean sink organs receive photosynthates from multiple sources through well-separated vascular pathways. Since phloem translocation includes not only sugars but also metabolites, amino acids, hormones, ions, and mRNAs, this work contributes to a broader understanding of plant physiology and may ultimately help improve nutrient allocation and crop productivity.
